# Prediction of Local Ultimate Strain and Toughness of Trabecular Bone Tissue by Raman Material Composition Analysis

**DOI:** 10.1155/2015/457371

**Published:** 2015-01-28

**Authors:** Roberto Carretta, Edgar Stüssi, Ralph Müller, Silvio Lorenzetti

**Affiliations:** Institute for Biomechanics, ETH Zurich, Vladimir-Prelog-Weg 3, 8093 Zurich, Switzerland

## Abstract

Clinical studies indicate that bone mineral density correlates with fracture risk at the population level but does not correlate with individual fracture risk well. Current research aims to better understand the failure mechanism of bone and to identify key determinants of bone quality, thus improving fracture risk prediction. To get a better understanding of bone strength, it is important to analyze tissue-level properties not influenced by macro- or microarchitectural factors. The aim of this pilot study was to identify whether and to what extent material properties are correlated with mechanical properties at the tissue level. The influence of macro- or microarchitectural factors was excluded by testing individual trabeculae. Previously reported data of mechanical parameters measured in single trabeculae under tension and bending and its compositional properties measured by Raman spectroscopy was evaluated. Linear and multivariate regressions show that bone matrix quality but not quantity was significantly and independently correlated with the tissue-level ultimate strain and postyield work (*r* = 0.65–0.94). Principal component analysis extracted three independent components explaining 86% of the total variance, representing elastic, yield, and ultimate components according to the included mechanical parameters. Some matrix parameters were both included in the ultimate component, indicating that the variation in ultimate strain and postyield work could be largely explained by Raman-derived compositional parameters.

## 1. Introduction

The mechanical properties of trabecular bone tissue have been extensively investigated in the past [1–9]. In these efforts, computed tomography has become an important tool when combined with testing of trabecular bone because this approach allows for relating apparent mechanical properties to bone mass and morphology [10]. One of the major clinical applications to study bone is dual X-ray absorptiometry (DXA), which is the current standard tool for the estimation of fracture risk and diagnosis of osteoporosis, as defined by the World Health Organization [11, 12]. Nevertheless, clinical studies indicate that although bone mineral density correlates with fracture risk at the population level, it does not correlate at the individual level [12–14]. Thus, bone mass alone has limited utility as an effective and precise indicator for diagnosis and clinical treatment of bone. Moreover, the etiology of any type of fracture among older adults is multifactorial, involving many extraskeletal risk factors [15] difficult to take into consideration as well as intraskeletal parameters (such as muscle attachment, bone shape and architecture, fabric orientation, material properties, and remodeling) not yet fully investigated. Considering the aging population, there is a definite need for better predictive tools. Current research aims to develop better predictive tools based on the available clinical data and to better understand the failure mechanism of bone, which can lead to the identification of key determinants for improving fracture risk prediction. In this work, we focus on trabecular bone, which may provide the dominant mechanical properties and play an important role in osteoporotic fractures, such as in vertebrae, but, in general terms, this idea is still a matter of ongoing scientific debate [16–18].

It is often postulated that various factors must be considered to play a role in the assessment of the mechanical properties of trabecular bone tissue, with bone mass playing an important but not unique role [19–22]. A variety of determinants have been introduced thus far, from the macroscale down to the nanoscale, to better characterize bone tissue, also commonly referred to as “bone quality.” At the macroscale, the architecture of trabecular bone can be measured using microcomputed tomography (micro-CT), allowing for the assessment of morphological indices and the fabric of the tissue [21]. At the tissue level, tissue turnover and remodelling can also be evaluated by means of micro-CT, quantitative back-scattered electron scanning microscopy (qSEM), and classical histology [23–25], while synchrotron radiation allows for the detection and analysis of microdamage [26, 27]. At the nanoscale atomic force microscopy can be used to measure factors such as the size of bone mineral grains. The material composition has been investigated using high-performance liquid chromatography (HPLC) [28], Fourier transformed infrared spectroscopy (FTIR) [29, 30], and Raman spectroscopy [31, 32], a technique which requires less effort in sample preparation with respect to FTIR and allows one to assess collagen concentration, mineral-to-organic ratio, and cross-link typology. Although these determinants, together with bone mass, could potentially describe or even predict the mechanical competence of bone, the connections between these factors are still unknown, as is the effect of each factor on the final outcome.

Within this broad research field, the present work focuses on the specific questions of whether and to what extent the mechanical properties are correlated with material properties at the tissue level. We hypothesized that, beyond the elastic limit of the bone tissue, the structure of the collagen fibres and the bone matrix composition have a significant and measurable effect on the ultimate mechanical properties of single trabeculae. Furthermore, we aimed at disclosing the underlying and common sources of variation for the variables investigated.

## 2. Materials and Methods

### 2.1. Sample Mechanical and Material Properties

The mechanical and material data were acquired in a previous study [33] using a validated system for tension and bending tests of single trabeculae [34], which showed an accuracy error of 0.3% and a precision of 2.7%. Briefly, the methodology involved the preparation of samples, the acquisition of the material properties and geometry of each sample, the mechanical testing, and the use of finite element analysis (FEA) to back-calculate the tissue-level stress-strain curves. The material source consisted of two human femoral metaphyses (ethical approval reference number: EK-29/2007). The donors were both female and not pharmacologically treated; one (56 years at extraction) was clinically classified as healthy, and the second (54 years at extraction) exhibited secondary osteoporosis (FRAX, WHO Fracture Risk Assessment Tool). Based on the diagnosis, two groups were created (healthy and osteoporotic donor); within each, 2 subgroups were created based on testing mode (tensile and bending), resulting in a total of 4 groups of 8 samples each. Trabeculae were taken from the same anatomical location between the donors and aligned along the femur neck axis. The trabeculae were typically 400–600 *μ*m in length with a diameter of 100–180 *μ*m. Nominal dimensions of the trabeculae in the different groups were equivalent. Thirty-two samples were isolated in total (8 for each of the four subgroups), and one sample was not tested due to complete embedding during the preparation. One sample was removed from the analysis due to a data recording error.

**Figure 1:**
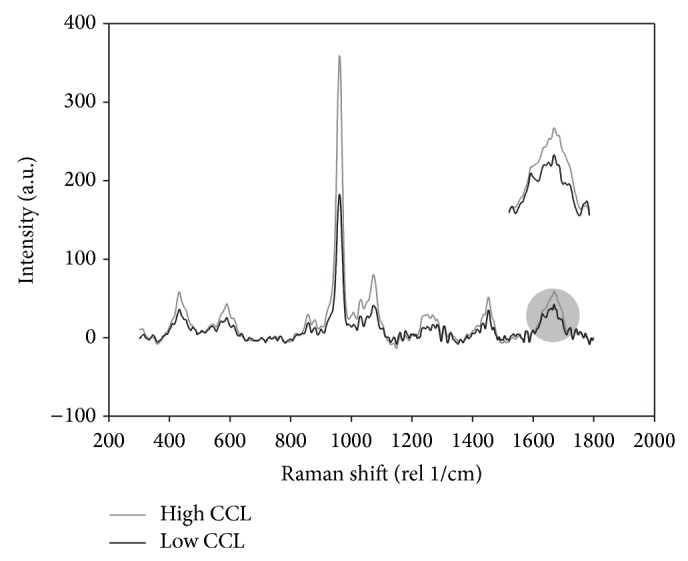
Typical Raman spectra for two random samples. The magnification on the right highlights the different high and low values for collagen cross-link (CCL) for the two samples.

A density-calibrated micro-CT system (*μ*CT 40, SCANCO Medical AG, Brüttisellen, Switzerland) with a nominal resolution of 6 *μ*m (energy: 55 kVp, intensity: 145 *μ*A, integration time: 200 ms, frame averaging: 3x, Gaussian filtration: *σ* = 1.2, and threshold: 330 mgHA/cm^3^) was used to calculate the local average tissue mineral density (TMD) and to create input meshes for FEA based on fixed density-based threshold. Prior to mechanical testing, the material composition was analyzed by means of Raman spectroscopy (Confocal Raman Microscope, CRM-200, WITec Wissenschaftliche Instrumente und Technologie GmbH, Ulm, Germany), in order to measure the structure of the collagen fibers and the bone matrix composition. A green laser source (wavelength of 532 nm) was focused with a 20x/0.4 NA objective. To compensate for the local variability of the tissue composition, a total of 10 measurements were taken from random locations on the surface of each trabecula [33]. Data measured was not sensitive to orientation. The single spectra were smoothed with a three-point moving average filter, and the fluorescent background was removed with a fifth-order modified multipolynomial fitting procedure [35, 36]. The material parameters were extracted based on the maximal peak intensity. The data clearly indicated that the amide I signal was characterized by two main peaks at 1632 cm^−1^ and 1663 cm^−1^ [33, 34] (Figure 1). These bands are emerging from amide vibrations of the backbone; they reflect secondary structure of the protein and are expected to be affected by cross-links [37], even though no conclusive validation has been performed for Raman spectroscopy yet, as for FTIR [29]. What can be assumed is that this parameter is an indication of collagen quality; it has been named collagen cross-links (CCL). A correlation was shown between a reduction in the postyield work and an increase in the ratio between the two subpeaks in the amide I region [31]. In that case, bands corresponding to immature and mature cross-links were observed at 1610 and 1655 cm^−1^ and were acquired with UV light. These bands were not observed in our data, which was obtained with a different laser wavelength (532 nm), but the subpeak at 1663 cm^−1^ and 1632 cm^−1^ in the Amide I bad was clearly visible instead. From the collected spectra, the following parameters were extracted: the mineral-to-matrix ratio (MMR, phosphate PO_4_^3−^ *ν*_1_ peak over amide I peak), B-type carbonate substitution ratio (CPR, carbonate CO_3_^2−^ *ν*_1_ peak over phosphate PO_4_^3−^ *ν*_1_ peak), and the collagen cross-link ratio (CCL, amide I subpeak at 1663 cm^−1^ over amide I subpeak at 1632 cm^−1^).

The mechanical testing was performed in dry condition on a custom-made stage equipped with a three-axis manual positioning device. The stage was fixed on a CLSM confocal microscope (LSM 510, Carl Zeiss MicroImaging GmbH, Germany). A rounded loading tip (radius = 0.1 mm) and two supports (span lengths = 0.53 mm) were used for the three-point bending test. The tensile tests employed a custom clamping mechanism at the embedded extremities of the trabecula, which allowed for the alignment of the trabecula by means of a high-magnification digital camera (Dino-Lite AD7013MTL, AnMo Electronics Corporation, Hsinchu, Taiwan) and for the application of the force along its axis. A stepwise strain-locked loading protocol was implemented, and after the measurement of the dimensions of the samples, each load step was estimated to apply a maximum strain step of approximately 0.2%, with an estimated constant strain rate <0.001 s^−1^, in order to eliminate the effect of creep. Each sample was loaded until failure. The surface strain at each loading step was optically measured by acquisition of a three-dimensional stack of the trabecular surfaces where a random pattern of fluorescent microspheres (diameter: 6 *μ*m, Fluoresbrite Yellow Green (YG), Polysciences, Inc., Warrington, Pennsylvania, USA) was deposited [38]. Finite element analysis implementing a Voce exponential nonlinear model [39] was subsequently used to calculate the intrinsic mechanical properties of each sample. The analyzed parameters were the elastic modulus *E*, yield stress *σ*_*y*_ and strain *ε*_*y*_, ultimate stress *σ*_*u*_ and strain *ε*_*u*_, elastic work (EW), and postyield work (PYW).

### 2.2. Statistical Models

All statistical tests were conducted with SPSS Statistics 20 (IBM SPSS Statistic, Version 20, IBM Corporation, New York, USA).

Linear regression analysis was implemented to identify the statistically significant relationships and to verify whether the structure of the collagen fibers and the bone matrix composition have a significant relationship with ultimate properties. A total of 4 material parameters and 7 mechanical parameters were analyzed [33], and the tensile and bending groups were analyzed separately. In both groups, the relationship between the mechanical and material parameters was evaluated with linear regression analysis for the two donors separately. A covariate model was applied to test whether the two regression slopes and intercepts of each donor were significantly different, in both tensile and bending groups.

Hierarchical multivariate regression analysis was used to investigate the extent to which each of the material parameters from both donors independently contribute to the variance in the ultimate properties. The ultimate strain was used as the dependent variable, and TMD, CPR, CCL, and MMR were entered stepwise as independent variables, beginning with the variable with the highest Pearson correlation coefficient. The model included all variables that were significantly correlated with the dependent variable.

Principal component analysis was run on the entire dataset to investigate common sources of variation for the mechanical and material properties and to identify whether they can be grouped or separated into components that explain the total variance of the data. Kaiser-Meyer-Olkin measure for sampling adequacy and Bartlett's sphericity tests were conducted to verify the appropriateness of this analysis. The Direct Oblimin (Delta 0) rotation method was used, and the component correlation matrix was evaluated to identify possible associations between the extracted components.

## 3. Results

**Figure 2:**
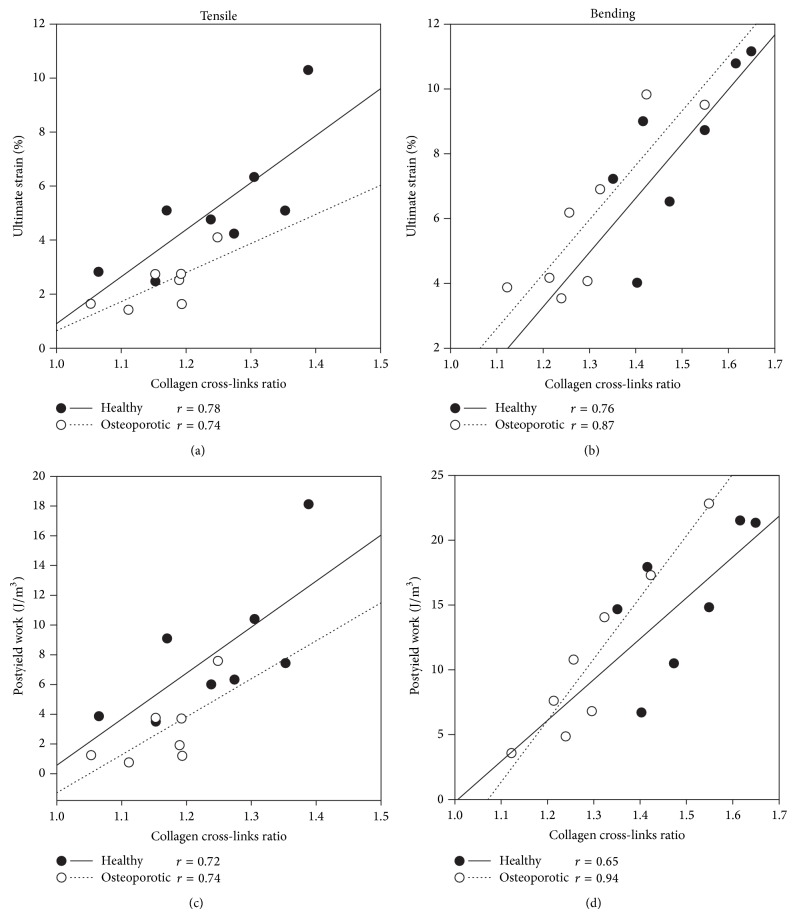
Regression analysis for the two donors showing the ultimate strain and the postyield work versus the collagen cross-link ratio ((a)–(c) for tensile and (b)–(d) for bending data, resp.). Tensile and bending data are analyzed separately and the Pearson correlation coefficient is reported for each regression line (healthy donor: solid line; osteoporotic donor: dotted line). *P* values for all correlation coefficients are in the range of 0.011–0.048.

**Figure 3:**
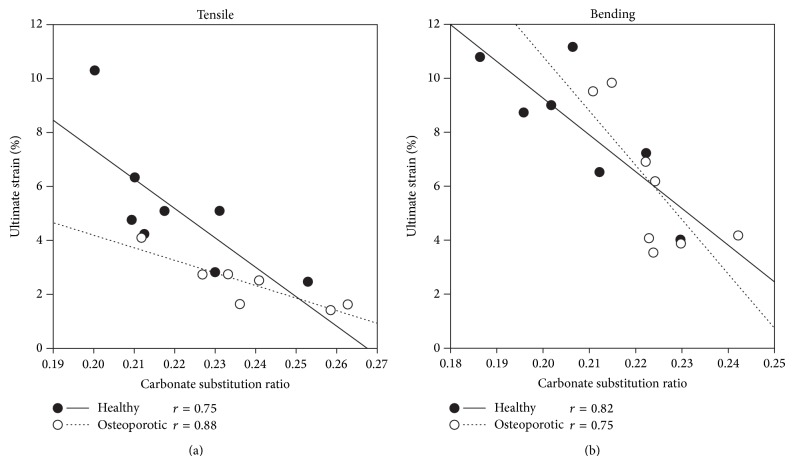
Regression analysis for the two donors showing the ultimate strain (a) and the postyield work (b) versus the carbonate substitution ratio. Tensile and bending data are analyzed separately and the Pearson correlation coefficient is reported for each regression line (healthy donor: solid line; osteoporotic donor: dotted line). *P* values for all correlation coefficients are in the range of 0.005–0.047.

For each donor in both bending and tensile groups, tissue mineral density, mineral-to-matrix ratio, and total amount of collagen (calculated from MMR and TMD) were not significantly correlated with any of the mechanical parameters. The carbonate substitution and collagen cross-link ratios were only weakly and nonsignificantly correlated with all of the mechanical parameters but strongly correlated with the ultimate strain and postyield work (Figures 2 and 3; *P* values for all correlation coefficients are in the range 0.011–0.048). Additionally, CPR and CCL were negatively correlated. The regression lines of ultimate strain and PYW versus CPR and CCL (Figures 2 and 3) were statistically compared for each donor in both tensile and bending groups, and no significant difference in slope and intercept was found. Post hoc statistical power for all regressions was evaluated and found to be larger than 0.898.

Multivariate regression analysis was performed using a stepwise method that included all of the factors that produced significant correlation with the dependent variables. We used TMD, CCL, CPR, and MMR as independent entries and the ultimate strain as the dependent variable. The results of the multivariate regression analysis indicate that the first variable in the model was CCL, with an adjusted *R* ^2^ of 0.625 for the tensile group and 0.689 for the bending group (Table 1). The multivariate regression analysis on the tensile group also included CPR as a second variable and the model could explain 73% of the total variance (Table 1).

Using a multivariate linear regression model including all the independent variables TMD, CCL, CPR, and MMR, the ultimate strain could be estimated based on the following equations:

(1) εu=13.68+10.27·CCL∗−69.16·CPR∗ −0.00·TMD−0.67·MMR,

(2) εu=27.49+6.95·CCL∗−126.57·CPR −0.00·TMD−0.27·MMR,

where “^*^” indicates the independent variables significantly contributing to the model. Equation (1) refers to tensile loading and (2) refers to bending loading.

**Figure 4:**
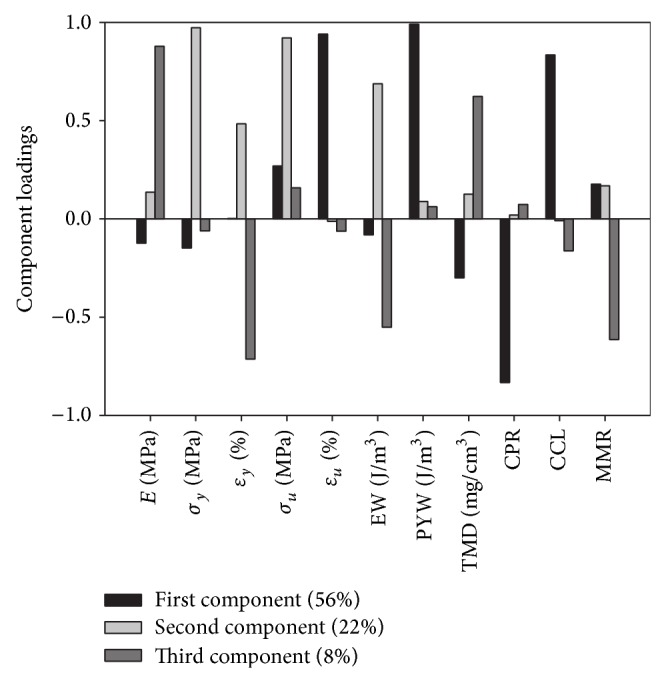
Principal component analysis showing the loading for the first 3 extracted components with the amount of total variability explained by each one. Included variables: elastic modulus *E*, yield stress *σ*_*y*_ and strain  *ε*_*y*_, ultimate stress *σ*_*u*_ and strain *ε*_*u*_, elastic work (EW), postyield work (PYW), tissue mineral density (TMD), collagen cross-links (CCL), carbonate substitution ratio (CPR), and mineral-to-matrix ratio (MMR).

Principal component analysis identified three main components that explain 86% of the total variance. The weight of the variables on each component (component loading) is reported in Figure 4. The first component, which accounts for 58% of the total variance, is associated with the inelastic response (high loading on ultimate strain and PYW) and includes both CCL and CPR, which can be independently associated with the ultimate mechanical properties of bone. The second component, which accounts for 22% of the total variance, includes yield stress, yield strain, and elastic work but none of the material parameters. The second component also has a high loading for ultimate stress, which is correlated with yield stress [33], but it is not correlated with ultimate strain or PYW. A third component, with a low explained variance (8%), includes high loadings for the elastic modulus, yield strain, elastic work, and tissue mineral density, which makes it likely to be associated with the elastic behaviour of bone. The analysis of the component correlation matrix shows that none of the components are correlated with each other. Each mechanical variable is present with high loading in only one component, which allows naming them the elastic, yield, or ultimate component, indicating that they may independently explain different aspects of the bone mechanics.

## 4. Discussion

The implemented experimental design allowed us to measure material composition properties together with mechanical properties at the level of single trabeculae. The key advantage of the individual mechanical testing of single trabeculae is that it allows analyzing tissue-level properties excluding the influence of macro- or microarchitectural factors (such as bone geometry) and better understanding the correlation between bone strength and material composition.

By means of Raman spectroscopy and micro-CT, we could measure the structure of the collagen fibers (CCL), the bone matrix composition (CPR, MMR), and the tissue mineral density (TMD). We used the information gathered to evaluate our hypothesis that, beyond the bone tissue elastic limit, the structure of the collagen fibers and the bone matrix composition have a significant and measurable effect on the ultimate mechanical properties.

For the first time, it was demonstrated that, at the microscopic level, ultimate strain-related but not stress-related mechanical properties have a strong relationship with collagen structure and matrix organization. A similar conclusion was reported at the organ level while investigating the effect of exercise on skeletal fragility [40] and it showed an increase in the CCL, CPR, ultimate strain, and toughness when testing mouse tibiae subjected to exercise. At the donor level, two studies reported a trend towards a higher but not significant CPR in fractured trabecular bone as observed in osteoporotic patients [41, 42]. The reported results can be thought of as an effect of what has been proved here at the tissue level: a higher B-type carbonate substitution ratio affects the mineral phosphate matrix in such a way that it correlates with ultimate strain and PYW, ultimately increasing the risk to fracture. The relationship between CCL and mechanical parameters has been investigated in other studies focusing at different scales, often producing contrasting results. It must be noted that our parameter CCL is measured by Raman spectroscopy and represents collagen quality, while no conclusive validation has been performed yet on the measurement of mature-immature cross-link with this technology. In cortical bone, an increase in the mature cross-links caused by high-temperature incubation and measured with HPLC was associated with a decrease in the ultimate strain and a decrease in the PYW [43], even though no information was obtained on immature cross-links and therefore on collagen fibers structure (CCL). In another study [44], at the tissue level, the tensile ultimate strain of single trabeculae regressed with the cross-link content showed no significant correlation with the absolute values of mature cross-links. Even though a direct comparison cannot be made due to the different scale, at the donor level, mechanical and biochemical tests on human vertebrae indicated that the ultimate strain was significantly and positively correlated with mature and not with immature cross-links [45], similar to what was also disclosed in a previously mentioned study [40], where an increase was shown in the CCL and CPR, as well as in ultimate strain and toughness at the organ level. In general, it can be concluded that the collagen fibers structure (CCL) has an effect on ultimate strain and PYW, even though a univocal relationship has not been proven yet. To critically evaluate this data, it must be considered that multiple factors are simultaneously responsible for the ultimate properties of bone. Therefore, it is likely that the discrepancy in the data reported in the literature may be due to the different scales of investigations and different testing setup, for example, organ-level mechanical properties combined with the average ultrastructural material properties. Our testing protocol measures the mechanical properties at the microlevel, thus reducing this gap, and our data shows that the CPR and CCL are significantly correlated with the tissue-level ultimate strain and PYW. The results are supported by other studies both at the organ [40, 45] and donor [41, 42] levels, even though further investigation should be performed to understand how strong the relationship between material and mechanics at the tissue level and at the donor level is.

Given the proven correlation between material properties and ultimate strain, we used this data to provide a statistical model of this relationship (see (1) and (2)), including all the material parameters. The results of the hierarchical multivariate regression analysis indicated that a single variable, CCL, has a strong predictive power (adjusted *R* ^2^ of 0.63 for the tensile group and 0.69 for the bending group (Table 1)). Additionally, the multivariate regression analysis on the tensile group also included the CPR as a second variable and could explain 73% of the total variance. For the first time, we reported that both CPR and CCL contribute separately to explaining the variance in the ultimate strain, at least for the tensile group, and that alone can explain a significant amount of the total variance. Nevertheless, given the high correlation between the two parameters and the limited effect of CPR on the hierarchical regression model, it is likely that these two factors share a common source of variation and could possibly be triggered by the same biological mechanism.

The second aim of our work was focusing at disclosing whether the underlying sources of variation were present to cluster and model the data. Principal component analysis appeared to be able to separate the mechanical outcome in three different regions: elastic, yield, and ultimate component. For the first time, we showed that these factors can be isolated, since the components were not correlated with each other. Each component therefore independently explains a different aspect of bone mechanics and can be separately used for bone modeling: the elastic component, characterized by elastic modulus and TMD, the yield, and the ultimate component. The latter is characterized by ultimate mechanical properties which vary together with the quality of the structure of the collagen fibers and the bone matrix composition.

These results suggest that modeling of bone failure needs to be a function of both strain and quality of collagen and mineral structure. From a mechanical point of view, it is likely that a strain-based model could be the most appropriate to describe tissue failure, as suggested in other studies on cortical bone where a strain-controlled failure was proposed [46]. Unfortunately, the limited number of donors does not permit us to extrapolate a generally valid model. Nevertheless, few important notes can be used for further investigation and possible implementation. For both the tensile and bending test modes, the regression slope and the intercepts of the ultimate strain and postyield work versus the carbonate substitution ratio and collagen cross-link ratio are not significantly different for the two donors, indicating that the same regression model is appropriate. The two donors (osteoporotic and healthy) can anyway be separated by evaluating CCL and CPR and by ultimate strain [33], even though it has yet to be proven whether this is true on a larger scale. Nevertheless, with the currently available data, the reported regression model could be possibly used to estimate a patient-specific ultimate strain or PYW from mean CCL and CPR. If this conclusion could be statistically supported, this approach would allow implementing the results of the regression model as a part of a constitutive model when predicting patient-specific bone failure.

Certain limitations in this work must be addressed. First, our results are limited to the microscale only. The advantage of a direct comparison between the material and mechanical properties is counterbalanced by the difficulty in extrapolating to more general results at the macroscopic scale, since single trabeculae are not isolated entities in physiological conditions. In this case, multiple factors acting on different scales are responsible for the high physiological variability in bone mechanics and must therefore be considered for an exhaustive and realistic characterization of bone mechanics [18] but can hardly be included or evaluated in the same study. Furthermore, extension to other mechanical real-case loading scenarios (e.g., the impact test) is important because failure often occurs in such circumstances. The use of only 2 donors is another limitation of this study; also no donor with atypical fractures was included. Additional research with more individuals is needed for the validation of the model capable to predict bone mechanical behavior, particularly when comparing healthy and osteoporotic donors. A major limitation to this research is that the presented methodology offers limited implementation in vivo. Consequently, the acquisition of these data for use in predictive analysis is limited, although a measure for the CCL and CPR may be obtained by transcutaneous measures in the future [47]. An additional aspect to be noted is that samples were tested in dry condition, which is only partially reflective of in vivo conditions. Nevertheless, even though absolute values will most likely be different for dry in vitro and wet in vivo values, the main conclusions of this work are drawn from correlation and PCA analyses, limiting the effect due to testing environment.

## 5. Conclusion

To test our hypothesis that beyond the elastic limit of the bone tissue the structure of the collagen fibers and the bone matrix composition have a significant and measurable effect on the ultimate mechanical properties, the entire set of mechanical and material parameters was evaluated using linear regression analysis and hierarchical multivariate regression analysis. For the first time, it is reported that both carbonates substitution ratio and the collagen cross-link ratio are two important predictors of ultimate strain and PYW, since they highly correlate with those parameters and they contribute independently to explain a significant amount of the variance in the ultimate strain and PYW. In respect to the clinical importance of bone material composition, these findings indicate that the mineral quality and the collagen organization play a dominant role in the determination of the local failure resistance of trabecular bone tissue and not the absolute value of mineral density or collagen concentration.

Even though the limited number of donors does not permit the extrapolation of a generally valid model in this pilot study, it suggests that the reported regression model could be used to estimate a patient-specific ultimate strain or PYW from means CCL and CP. Another important finding is that, by means of principal component analysis, the material and mechanical properties were clustered into three independent regions, corresponding to the elastic, yield, and ultimate mechanical behavior. The latter also includes CCL and CPR, suggesting that the reported regression model could be implemented as part of a constitutive strain-based model when predicting patient-specific bone failure. Further investigation is needed to statistically support this conclusion and refine the statistical model.

## Figures and Tables

**Table 1:** Results of the hierarchical multivariate regression analysis with the ultimate strain as the dependent variable and density (TMD), collagen cross-links (CCL), carbonate substitution ratio (CPR), and mineral-to-matrix ratio (MMR). Model 2 for tensile data and Model 1 for bending data include all variables that significantly contribute to the regression analysis.

	Tensile		Bending
	Model 1 (CCL)	Model 2 (CCL, CPR)	Model 1 (CCL)
Included variables	CCL	CCL, CPR	CCL
*R*	0.808	0.876	0.843
Adjusted *R* square	0.625	0.728	0.689
Standardised beta	0.808	0.505, −0.455	0.843
Excluded variables	CPR, TMD, and MMR	TMD, MMR	CPR, TMD, and MMR
