# Corrigendum to “Prediction of Local Ultimate Strain and Toughness of Trabecular Bone Tissue by Raman Material Composition Analysis”

**DOI:** 10.1155/2017/9857302

**Published:** 2017-11-21

**Authors:** Roberto Carretta, Edgar Stüssi, Ralph Müller, Silvio Lorenzetti

**Affiliations:** Institute for Biomechanics, ETH Zurich, Vladimir-Prelog-Weg 3, 8093 Zurich, Switzerland

In the article titled “Prediction of Local Ultimate Strain and Toughness of Trabecular Bone Tissue by Raman Material Composition Analysis” [1], there was an error regarding the FRAX® tool, which should be clarified as follows.

The article notes, “the donors were both female and not pharmacologically treated; one (56 years at extraction) was clinically classified as healthy, and the second (54 years at extraction) exhibited secondary osteoporosis (FRAX, WHO Fracture Risk Assessment Tool).” However, the World Health Organization (WHO) did not develop, test, or endorse the FRAX tool or its recommendations [2]. The metabolic bone disease unit at the University of Sheffield that developed FRAX was a WHO Collaborating Centre from 1991 to 2010, but treatment guidelines must undergo a formal process before they can be endorsed by the WHO.
